# Multivariate Analyses and Classification of Inertial Sensor Data to Identify Aging Effects on the Timed-Up-and-Go Test

**DOI:** 10.1371/journal.pone.0155984

**Published:** 2016-06-06

**Authors:** Danique Vervoort, Nicolas Vuillerme, Nienke Kosse, Tibor Hortobágyi, Claudine J. C. Lamoth

**Affiliations:** 1 University of Groningen, University Medical Center Groningen, Center for Human Movement Sciences, Groningen, The Netherlands; 2 University Grenoble-Alpes, AGEIS, La Tronche, France; 3 Institut Universitaire de France, Paris, France; Banner Alzheimer's Institute, UNITED STATES

## Abstract

Many tests can crudely quantify age-related mobility decrease but instrumented versions of mobility tests could increase their specificity and sensitivity. The Timed-up-and-Go (TUG) test includes several elements that people use in daily life. The test has different transition phases: rise from a chair, walk, 180° turn, walk back, turn, and sit-down on a chair. For this reason the TUG is an often used test to evaluate in a standardized way possible decline in balance and walking ability due to age and or pathology. Using inertial sensors, qualitative information about the performance of the sub-phases can provide more specific information about a decline in balance and walking ability. The first aim of our study was to identify variables extracted from the instrumented timed-up-and-go (iTUG) that most effectively distinguished performance differences across age (age 18–75). Second, we determined the discriminative ability of those identified variables to classify a younger (age 18–45) and older age group (age 46–75). From healthy adults (n = 59), trunk accelerations and angular velocities were recorded during iTUG performance. iTUG phases were detected with wavelet-analysis. Using a Partial Least Square (PLS) model, from the 72-iTUG variables calculated across phases, those that explained most of the covariance between variables and age were extracted. Subsequently, a PLS-discriminant analysis (DA) assessed classification power of the identified iTUG variables to discriminate the age groups. 27 variables, related to turning, walking and the stand-to-sit movement explained 71% of the variation in age. The PLS-DA with these 27 variables showed a sensitivity and specificity of 90% and 85%. Based on this model, the iTUG can accurately distinguish young and older adults. Such data can serve as a reference for pathological aging with respect to a widely used mobility test. Mobility tests like the TUG supplemented with smart technology could be used in clinical practice.

## Introduction

There is a growing interest in identifying an array of measurements that can assess relevant processes associated with healthy ageing (e.g., [1–5]. Such “biomarkers” can concurrently change with age but can also predict ageing-related phenotypes or subsequent health outcomes including morbidity, mortality, quality of life and health span. Measurements of biomarkers should be easy to administer and still provide clinically meaningful information as surrogate endpoints in interventions specifically designed to extend health span. Beyond interventions, population studies should also benefit from valid, reliable, low-cost indices of healthy ageing [3]. In general, biomarkers comprise key bodily functions, which are known to decline during ageing. Biomarkers should thus target physical capability and cognitive, physiological, musculoskeletal, endocrine and immune functions. Within the domain of motor function in aging, thanks to its high construct and convergent validity, reliability, and standardization the Timed-Up-and-Go (TUG) test has recently been proposed [4] and recommended as a potentially useful biomarker of healthy ageing [3]. The TUG is routinely used as a composite test to assess leg strength (sit-to-stand), gait, and balance (180° turn; sit-to-stand, stand-to-sit). Constituent elements of TUG represent activities of daily living linked to quality of life in healthy aging. Unsurprisingly, TUG has hence become a popular and informative mobility test that provides age-, gender-, and pathology-specific data on old adults’ balance and gait function [6, 7]. Even though a stopwatch is sufficient to assess TUG performance [8], total time as a summary measure cannot characterize the execution quality of its sub-phases. Such an omission is unfortunate considering that the postures and the transitions between phases of TUG are frequently administered as individual tests for the quantification of dynamic balance, walking ability [9], the capacity to sequence tasks [10], and even to assess fall risks [11]. Miniaturization, low weight, inconspicuousness, validity, reliability low cost, and versatility of automated algorithms to analyse a variety of motor tasks have made such devices the tool of choice for an objective quantification of motor function aging. Such sensor features make it possible to use wearable technology not only in a research setting but also in a clinical setting where individuals execute motor tasks in their natural environment [12, 13].

Inertial measurement units (IMU’s) with embedded 3D accelerometers and gyroscopes can quantify key phases of the instrumented TUG (iTUG) and provide in-depth information on functional performance [14–16]. Algorithms such as Hidden Markarov Models [17], Dynamic Time Warping [18], and methods for dimensionality reduction can characterize temporal features of transition between phases of iTUG [19]. An automated detection of sub-phases of the iTUG can characterize movement in terms of smoothness, regularity, variability, maximal velocity, or range in angular velocity. Phases of iTUG are sensitive and can classify frail [20] versus healthy elderly [21, 22] and identify those with fall risks [23], cognitive impairment [24], and assess stages or quantify movement impairments in Parkinson’s disease [25–27].

The use of iTUG is complicated by the difficulty in selecting from the large number of variables those that are sensitive to individual differences in gait and balance performance. Frequently used variables include the mean, median, standard deviation and ranges of a signal characterizing sub-phases of the iTUG. In addition, measures related to variability (RMS), smoothness of performance (Jerk/slope), gait variability index (Phase variability Index, Harmonicity Ratio, Coefficient of Variation of stride times) have been suggested for quantifying performance during specific iTUG phases [10, 14, 19, 23]. Most studies focused on distinguishing patients from healthy (older) adults. Moreover, the large number and variety of variables makes it difficult to determine the variables that could separate age groups of healthy adults over the lifespan. Pattern recognition methods like Principal Component Analysis (PCA) are suitable to gain insights into data matrices and minimize redundancy. Palmerini [19] applied PCA to search for a subset of variables relevant for three phases of the TUG, sit-to-stand, walking and stand-to-sit. Of the initial 28 variables of healthy adults based on accelerometer signals embedded in a smartphone, a reduced set of twelve variables was extracted using PCA, but these analyses were not used to stratify participants by age and the device also operated without a gyroscope.

iTUG has previously been used for patient stratification. A linear discriminant analysis of iTUG data stratified nearly 80% of healthy and early-mild Parkinson’s patients correctly, based on mediolateral (ML) and vertical Jerk during turning and anterior-posterior root mean square (RMS) during the sit-to-walk phase [25]. As compared with TUG duration measured with a stop watch, a binary logistic regression analysis of a subset of three variables (jerk of the sit-to-stand, average step duration, standard deviation (STD) of the overall performance) was more accurate in classifying non-fallers and fallers [23]. A pattern matching k-NN algorithm was also effective in distinguishing old adults with a low and high fall risk based on the RMS of the vertical acceleration during walking, the amplitude of the yaw signal during turning and the time to complete the test. Sit-to-stand and stand-to-sit related variables were not included in the classification [28].

Overall these studies show that a subset of parameters of the iTUG could classify certain type of patients. The current and sporadic evidence for using iTUG as a classification tool could be generalized and broadened by providing a normative database that characterizes a set of statistically selected variables for the postural and ambulatory elements of iTUG. Such data can then be used to assess the effects of natural aging and could serve as a basis for the identification of patients with mobility disability [10, 19, 23, 25]. Therefore, the first aim of our study was to identify iTUG variables that are associated changes in performances of the iTUG across the adult lifespan. Secondly after identification of the most important iTUG variables we assessed if these variables could accurately discriminate two age groups one of age 18–45 and one of age 46–75 year. Because the onset of decline in of muscle mass and muscle function starts around age 40–45 year, we chose a cut-off value of group division at age 45 [29–32]. We combined a wavelet analysis algorithm (to identify phases of iTUG) with a phase detection algorithm based on accelerometer and gyroscope data and applied statistical analyses to specify variables that could effectively classify healthy young versus old adults. To this aim, first we used a Partial Least Square analysis (PLS), a method that combines dimensionality reduction and regression, to identify the variables of the iTUG that are sensitive to age. Second, we examined the classification power of the identified variables to stratify young and old adults, using a PLS-discriminant analysis.

## Methods

### Participants

Fifty-nine healthy adults participated in the study (45±18 years, range of age: 18 to 75, 46% male) and served as a basis for two age groups: 18–45 (28±7 years, n = 28, 61% male; weight = 75.4 ±7.6 kg; length – 178±11.28 m) and 46–75 (62±8 years, n = 31, 32% male; weight = 73.1±13.3 kg; length = 169.5±9.4 m). All subject were healthy and active. Participants were asked to report the number of hours a week they engaged in physical activity during a typical week (e.g., tennis, dance, hiking, yoga). Participants in the younger group were on average 4.8±2.0 hours active a week and participants in the older group 2.9±2.2 hours a week.

Data of four participants (3 young; 1 old) was excluded from one of the two trials they performed, because the data was not correctly recorded due to a corrupt memory card. The local Ethical Committee of the Center of Human Movement Sciences of the University Medical Center Groningen approved the research proposal. All participants signed a written informed consent before participating. The iTUG test was part of a larger study examining the effects of age on gait [31].

### Instrumentation and procedure

Trunk accelerations were measured during the TUG with an Inertial Measurement Unit (IMU; DynaPort® hybrid unit (56x61x15 mm, 54 g; McRoberts BV, The Hague, the Netherlands). The unit consists of a tri-axial accelerometer and gyroscope sensor (100 Hz sample frequency). Data was stored on a SD card for off-line analysis of the signals. The IMU was fixed with an elastic belt at the level of lumbar segment L3 over the participant’s clothes. Participants performed the iTUG two times. The iTUG consisted of standing up from a chair without the use of the arms, walking 7 m, turning around a pion, walking 7 m back to the chair, and sitting down without the use of the arms. Participants were instructed to perform this task as fast as possible without running. Since the iTUG was performed in the context of a larger study the TUG trials were randomized with three other gait tests. All data analyses were performed off-line using Matlab software (version—R2015b, The MathWorks Inc.).

### Phase detecting algorithm

An algorithm was developed to detect five phases of the iTUG: 1) rising from a chair (sit-to-stand), 2) walking, 3) turning, walking, 4) turning and 5) sitting down (stand-to-sit) (see also [16, 22, 23, 33]). The two walking phases were pooled for gait analysis. Similar to the studies of Weis et. al., [22, 23] identification of postural transitions during sit-to-stand and stand-to-sit was based on the pitch of the angular velocity signal and on the anteriorposterior (AP) acceleration signal. Turns were identified from the yaw of the angular velocity signal [22, 34]. We used a discrete wavelet approach to perform a time frequency decomposition of the signals in order to identify the relevant signal peaks related to the start and end of phases of iTUG [33, 35–37] or the type of signals collected in the present study, a Daubechies (db) mother wavelet was appropriate [36, 38, 39].

### Standing-up and sitting down

**Fig 1:**
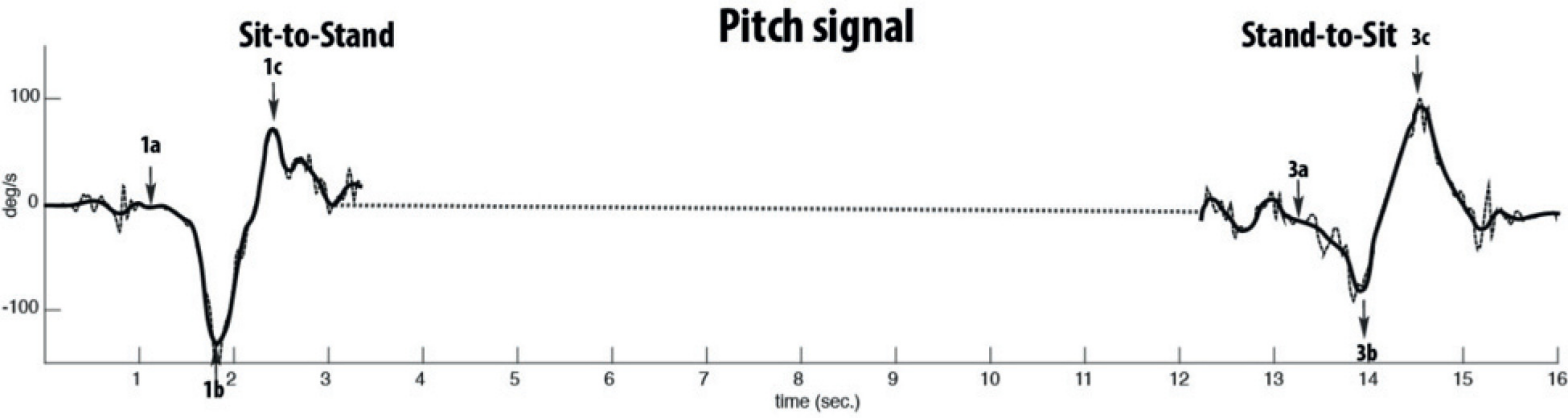
Representation of the Pitch signal for detecting standing-up and sitting down phases. Pitch signal or rotation around the mediolateral axis (dotted line) and reconstructed signal (solid line) using level 4 approximation of db5 wavelet. When the signal becomes negative (1a) the trunk moves forward until minimal angular velocity (1b). Subsequently when the participants stands-up the angular velocity also changes in direction. For sitting down the same pattern is visible (3a-3c).

The pitch signal was analysed with a db5 mother wavelet and its reconstruction was based on the level 4 approximation (4A). Thereafter, peaks (Fig 1) in the reconstructed signal were detected using a peak detection algorithm ‘findpeaks’ of the signal toolbox of Matlab, which searches for local maxima in the signal. Fig 1 presents the phases of standing-up and sitting-down. From 1a to 1b, the subject moves the trunk forward in preparation for rising from the chair. Subsequently, from 1b-1c the trunk is moved backward until standing upright. In the sitting down phase (3a-3c) the pattern is repeated.

### Turning

**Fig 2:**
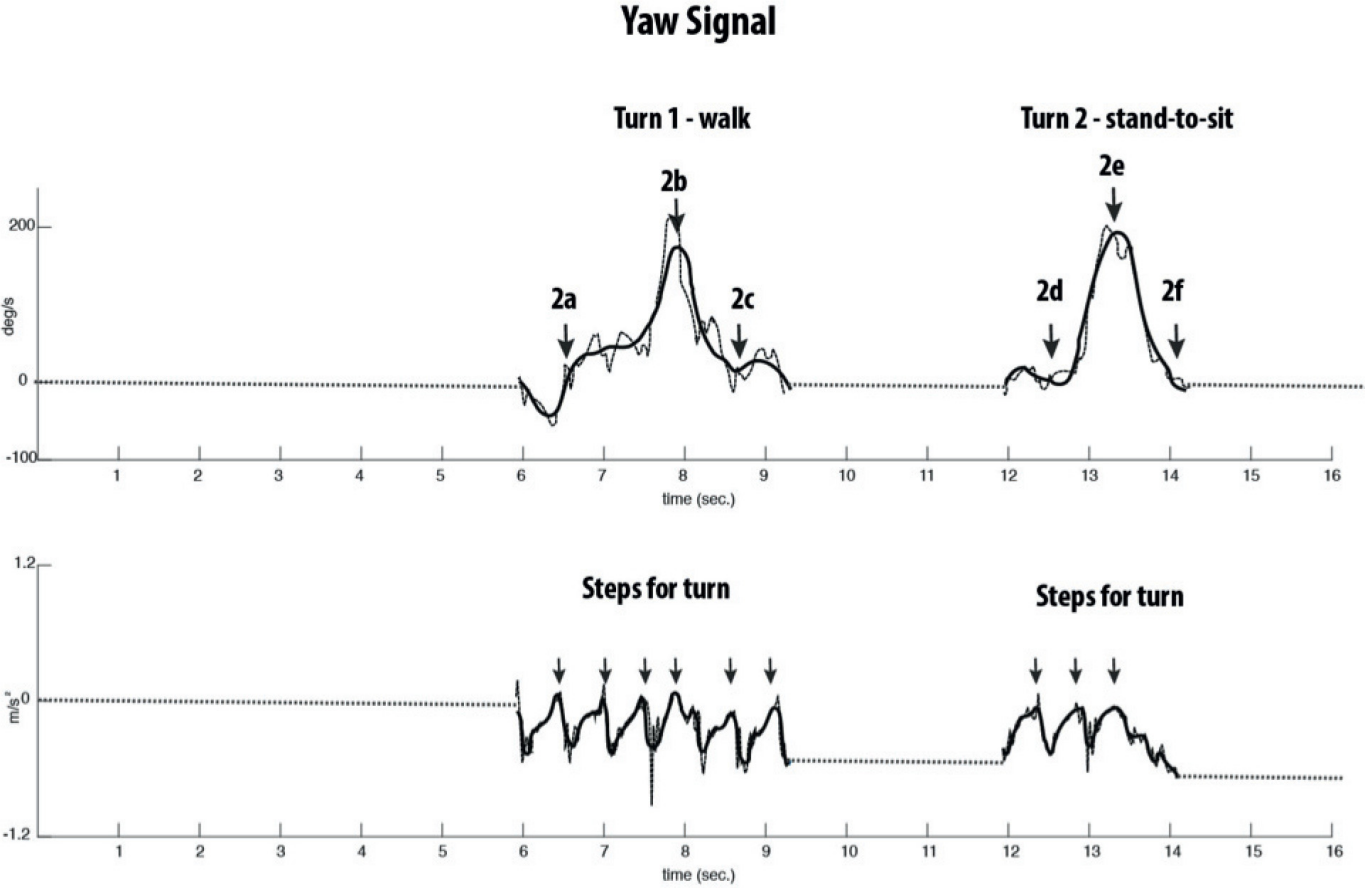
Representation of a yaw signal used for identifying the turn phases and of an AP acceleration signal for detecting steps during turns. The upper trace represents the yaw signal or rotation around the vertical axis (dotted line) and reconstructed signal (solid line) using a level 6 approximation of db5 wavelet. The turn is indicated by an increase/decrease in the yaw amplitude depending on the direction of the turn. Start of turning is when the zero line is crossed (2a, 2d) and end of turn when the zero line is again crossed (2c; 3f). The lower trace represents the AP acceleration signal (dotted line), reconstructed at level 3 with a db5 wavelet (solid line). Peaks indicate foot contact instances.

To detect the turn at the end of the first walking trajectory and before sitting-down, a db5 mother wavelet was used on the yaw signals and the reconstruction was based on the level 6 approximation (Fig 2). Depending on the direction of the turn a negative or positive peak appears. First, the minimum or maximum peak point in the wavelet is found (Fig 2, upper trace: 2B–2E). Thereafter, the first point where the yaw signal crossed the zero line is detected before and after the peak. This is done for both turns. To determine the number of steps used to turn, the trunk AP acceleration signal is reconstructed at approximate level 3 of db5 (Fig 2, lower trace). The peaks in the acceleration signal represent a foot contact instance.

**Fig 3:**
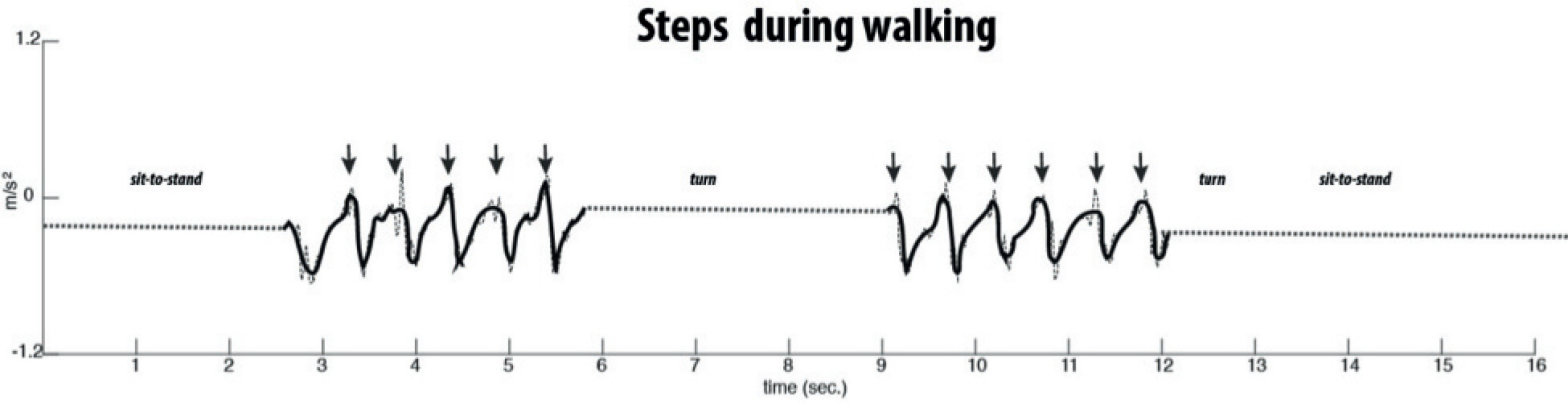
Representation of an AP acceleration signal for detecting steps during walking. The signal represents the raw (dotted line) and reconstructed (solid line) anterior-posterior acceleration signal (Level 3 db5), used for defining step parameters. Arrows indicate heel strike.

Detection of the start and end of the walking phases was based on the previous phases and foot contact moments extracted from the AP acceleration signal (Fig 3). The start of walk 1 was defined as the first peak after standing up (Fig 1 and 1C) and ended at the peak before the turn (Fig 2; 2A). The second walk after turning started at the first peak after the turn (Fig 2 and 2C) and ended at the peak just before the turn for sitting-down (Fig 2 and 2D).

### Variables calculated from the iTUG phases

We calculated the same variables for phases of iTUG that have been reported in the literature [10, 14, 19, 22, 23, 25, 28, 40]. First, the duration for each phase was calculated. Second, we calculated the amplitude, range of the movement, variability and smoothness of the movement for sit-to-stand, stand-to-sit, and for the two turns. Data for the two walking phases were combined. From foot contacts, step-related variables (e.g., stride time, number of steps) were calculated. From the ML and AP acceleration signals, we computed measures of stability and smoothness of gait. Altogether, we calculated 72 variables for the Partial Least Square (PLS) analysis (Table 1). Outcome measures were expressed in absolute values, being positive or negative signs according to the direction of turn.

**Table 1:** Variables calculated for different phases of the iTUG.

Variables	iTUG components*	Description	Signal / M.U.
Time	Sit–to-stand; Stand-to-sit; Turns; Walking	Duration of each phase	Sec.
Mean	Sit–to-stand; Stand-to-sit; Turns- Walking	Average value over different identified phases of iTUG	Pitch deg./s Pitch deg./s Yaw deg./s AP acc. m/s^2^
STD	Sit–to-stand; Stand-to-sit; Turns- Walking	Standard deviation calculated over identified phases of iTUG	Pitch deg./s Pitch deg./s Yaw deg./s AP acc. m/s^2^
Range	Sit–to-stand; Stand-to-sit; Turns- Walking	Difference between maximum and minimum observation	Pitch deg./s Pitch deg./s Yaw deg./s AP acc. m/s^2^
Max	Sit-to-stand; Stand-to-sit; turns- walking	Maximal value of the signal	Pitch deg./s Pitch deg./s Yaw deg./s AP acc. m/s^2^
Median	Sit-to-stand; Stand-to-sit; Turns- Walking	Middle value of signal values	Pitch deg./s Pitch deg./s Yaw deg./s AP acc. m/s^2^
RMS	Sit-to-stand; Stand-to-sit;	Root Mean Square:	Pitch deg./s Pitch deg./s Yaw deg./s AP acc. m/s^2^
	Turns- Walking	$RMS\sqrt{\frac{1}{N}}\sum_{i=1}^{N}{\left(x_{i}-\bar{\bar{x}}\right)}^{2}$	
		*x* = signal type	
Slope	Sit-to-stand St and-to-sit Turns	Rate of change in angular velocity, direction and steepness.	Yaw
N steps	Walking	Number of steps over the two walking tracts	n
Step time	Walking	Average time between right and left foot contact	AP acc. s.
CV step	Walking	Coefficient of Variation between steps	%
		$CV=\frac{\sqrt{\frac{1}{N}}\sum_{i=1}^{N}{\left(s_{i}-\bar{s}\right)}^{2}}{\bar{\bar{s}}}*100$	
		*s* = step time = signal, *i* = step number	
Phase deviation	Walking	*φ*_*i*_ = (*FCR*_*t*(*i*)_ − *FCL*_*ti*_)/(*FCL*_*t*(*i*+1)_ − *FCL*_*t*(*i*)_) * 360°	AP acc. unit less
		*φ*_*i*_ = Point-estimate of relative phase as measure of timing between contralateral heel strikes. FCR = time instant right heel strikes. FCL = left heel strike	
		${\bar{\bar{\varphi }}}_{dev}=\frac{1}{N}\sum_{i=1}^{N}\varphi_{i-}180$	
		Average deviation from perfect symmetric gait	
Phase variability	Walking	Because the relative phase is a circular measure, circular statistics was applied to calculate the variance of the relative phase over strides.	unit less
Index of harmonicity	Walking	$IH=\frac{p_{1}}{\sum_{i=1}^{10}p_{i}}$	AP – ML accunit less
		*p*_*i*_ = Power spectral density of fundament frequency	
		∑*p*_*i*_ = the cumulative sum of power spectral densitie of the 10 harmonics.	
		Higher IH indicates smoother gait pattern	
Gait cycle Variability	Walking	${SD}_{i}=\sqrt{\left(\frac{\sum_{j}^{N}{\left(x_{ij-}{Cycle}_{i}\right)}^{2}}{n-1}\right)}$	AP – ML acc unit less
		Point by point standard deviation for *i*^th^ sample s_*ij*_ signal value for *i*^th^ sample *j*^th^ step cycle *i* mean over cycle of *i*^th^ sample	
		$PhVar=\sqrt{\frac{\sum_{i=1}^{k}{{STD}_{i}}^{2}}{k}}$	
		Gait cycle var = average of individual point by point std values across all samples *k STD*_*i*_ standard deviation over *i*^th^ sample	
Frequency	Walking	1/ step time	AP – ML Acc Hz/s

*As indicated in Fig 1, sit-to-stand variables were calculated for phases 1b – 1c, 1a – 1c; for stand-to-sit from, 3a – 3b; 3a – 3c. Turn slope was calculated separately for phase 2a – 2b; 2b – 2c and 2d- 2e; 2e – 2f (see Fig 2) acceleration signal. AP = Anterior Posterior; ML = mediolateral; M.U. = Measurement Unit.

### PLS analyses

A Partial Least Squares (PLS) regression analysis was applied to determine the iTUG variables that were related to age (PLS-Toolbox 8.1 for Matlab, Eigenvector Research Inc.). PLS analysis combines PCA with regression analysis. Compared with step-wise regression or structural equation models, PLS methods can handle a larger set of independent variables with lower number of observations. Moreover, multivariate PLS regression allows the modelling of multiple responses, while dealing with multicollinearity [41], which is often present in motion data, including walking. The general aim of the PLS analysis is to define a maximum covariance model and explain the relationship between the iTUG variables (X-matrix, predictors) and age (Y-matrix, responses). In other words, successive orthogonal factors are chosen that maximize the covariance between each X-score and the corresponding Y-score to find a model that best predicts age with a selected number of iTUG variables.

Two separate PLS analyses were performed consecutively. For the first PLS analysis trial one was used as data input. With this data, a PLS model was built to determine the latent iTUG variables that most accurately predict age and also explains most of the covariance between iTUG variables and age. The second analysis consisted of a PLS-discriminant analysis (DA) to determine how accurately the iTUG variables identified by the first PLS analysis discriminate the two age groups.

The data were pre-processed by a z-transformation. For the first PLS analysis, the X-matrix consisted of the 72 iTUG variables and the Y-matrix the 57 participants’ age. By extracting the variables that contribute the most to the model, the number of variables is reduced to a smaller number of Latent Variables (LV). Any given LV explains a part of the total variance in the Y-matrix (age) by capturing the variance in the X-matrix (iTUG variables). The amount of variance of the iTUG variables explained by the models LV indicates the relevance of the variables in the prediction of age [41]. The number of LVs was determined by goodness of prediction (Q2).

$${Q2}_{k}=1-\frac{{PRESS}_{k}}{{RSS}_{k-1}}$$ (1)

$$PRESS=\sum {\left(y_{k-1,m}-{\hat{y}}_{k-1,-m}\right)}^{2}$$ (2)

where PRESS is the predictive sum of squares of the model containing *k* components and RSS is the residual sum of squares of the model[42]. The PRESS depends on the *y*_*k*−1,*m*_ the residual of observation *m* when k–1 components are fitted in the model and ${\hat{y}}_{k-1,-m}$ the predicted y when the latest observation of *m* is removed. When Q2 reaches a plateau, before it decreases, this is considered the optimal number of latent variables.

To assess the PLS model, several outcomes were derived. First, the goodness of fit (*R*2) of the model was determined. The *R*2 explains how well the model fits the data and is calculated as follows:

$${R2}_{k}=1-\frac{{RSS}_{k}}{TSS}$$ (3)

The *R*2 is defined by the residual sum of squares of the *k*^*th*^ LV and the total sum of squares (TSS). Next, the weights of the PLS model were assessed. They illustrate the relationship between iTUG variables and the participant’s age, with respect to the individual LV. The weights describe the importance of iTUG variables and age on the model for individual LV. If they are near zero for all identified LVs than they add little to the model.

To identify which iTUG variables are of importance to the model the regression coefficients of the PLS matrix and the Variable Importance for Projection (VIP) are evaluated. Whereas the regression coefficients (RC) represent the influence each variable has in the prediction of the response (age), the VIP represents the values of each predictor (iTUG-variable) in fitting the PLS model for predictors as well as the responses. A large absolute coefficient for an iTUG variable (predictor) together with a VIP values > 0.8 indicates that a variable is a prime candidate in the model [41].

The VIP scores are calculated as follows:

$${VIP}_{j}=\sqrt{p\sum_{k=1}^{N}\left[{SS}_{k}(w_{kj}/{\left\|w_{k}\right\|}^{2})\right]/\sum_{k=1}^{N}{\left(SS\right)}_{k}}$$ (4)

with *SS*_*k*_ is the explained sum of squares of the *k*^*th*^ *LV* and *N* the number of LVs in the model. Hence the *VIP*_*j*_ weights *w*_*kj*_ quantifies the contribution of each variable *j* according to the variance explained by each *k*^*th*^ *LV*. The selected variables were included in the second analysis PLS-DA.

### PLS-DA analysis

To determine the classification power of the iTUG variables identified in the first PLS analysis a PLS-DA was performed on the dataset of the second iTUG trial. The iTUG variables selected from the first PLS analysis thus formed the *X*-matrix. For the discriminant analysis, the participants were separated into two age groups, one with age 18–45 and one group with an age of 46–75 years.

Based on the PLS-DA a Receiver Operating Characteristic (ROC) curve was constructed. This curve includes both the true positive rate (sensitivity) and false positive rate (specificity) of the model. Each point on the ROC-curve represents a sensitivity/specificity pair, which is related to a threshold that determines the optimal boundary between younger and older adults in the classification. The Area Under the Curve (AUC) is an indicator of the classification power of the model. It is the average value of sensitivity for all possible values of specificity. An AUC of 1 shows a perfect accuracy of the classification and an AUC of 0.5 is a pure guess of the result.

## Results

### Phase detection of the iTUG

The PLS model contained three LVs, as the Q2 had reached a plateau at LV3 before it decreased. The three LVs explained 30.5% of the co-variance between the iTUG variables (X-matrix), and 71% of the variance in age (respectively explaining 49.4%, 9.9% and 11.7% of the variance in age).

**Fig 4:**
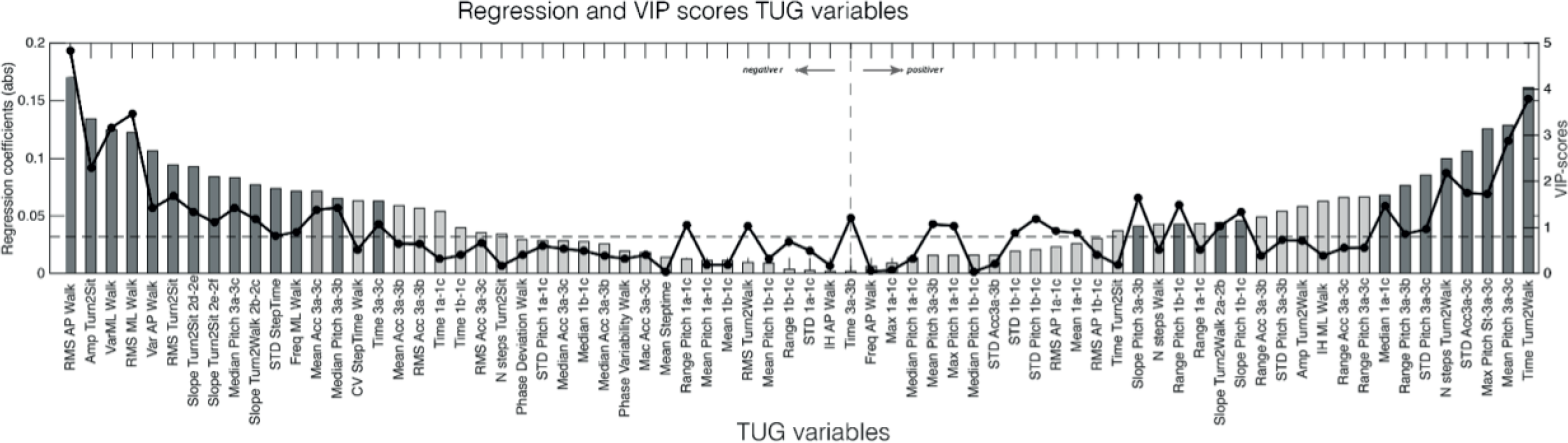
Variable Projection of Importance (VIP) scores and regression coefficient plot. The regression coefficients are giving as bars in absolute values. To the left and right of the vertical dotted line, respectively, the negative and positive regression coefficients are shown. The dotted black line represents the VIP-scores (right y-axis). In order to be important to the model, the dots in the dotted line should be above the dashed line (VIP > 0.8, right Y-axis). The dark bars are the variables that entered the PLS-DA model. Note that due to the large number of variable included in the model, regression coefficients are relatively low.

Fig 4 shows the VIP scores and absolute RC for all the iTUG variables included in the analysis. The variables on the left side are negative RC representing lower values of all included parameters except for stand-to-sit median pitch and mean acceleration in the 3a-3b phase. These values were related to participants with higher age. In addition, positive RC, on the right side, indicates that higher values on these variables are related to higher age. As illustrated in Fig 4, based on the criteria for selection of iTUG variables, (VIP score > 0.8 and RC > 0.04), 27 of the 72 iTUG variables were considered important to the PLS model. The 27 selected variables of the iTUG are related to different phases of the iTUG. Table 2 shows mean values, VIP scores, RC and the captured variance of each variable per LV.

**Table 2:** VIP (Variable Importance for Projection) and Variance captured by the 3 LV in the PLS model. Only variables with a VIP score higher than 0.8 are included. The means of the variables in the first dataset are also shown. Note that due to the large number of variables included in the model, regression coefficients (RC) are relatively low in this type of PLS models.

				Variance Captured			Young		Old	
Phase	Variable	RC	VIP Scores	L1	L2	LV3	Mean	STD	Mean	STD
Sit-to-stand	Range pitch (B-C)	0.04	1.49	40.2	33.6	0.3	157.05	34.45	191.46	51.04
	Slope pitch (B-C)	0.05	1.33	21.8	2.5	1.8	2.58	1.00	2.94	0.98
	Median AP (A-C)	0.07	1.46	27.2	15.2	0.1	0.47	0.13	0.53	0.13
Walk	RMS AP	-0.17	4.83	7.0	46.2	0.2	0.39	0.14	0.28	0.07
	RMS ML	-0.12	3.46	11.2	27.9	0.1	0.20	0.04	0.17	0.04
	Gait cycle variability ML	-0.12	3.17	8.4	26.5	0.1	0.19	0.04	0.17	0.05
	Gait cycle variability AP	-0.11	1.42	0.1	18.2	2.3	0.20	0.05	0.19	0.04
	STD step time	-0.07	0.81	4.9	0.2	3.9	0.08	0.03	0.07	0.03
	Gait frequency ML	-0.07	0.90	4.4	1	1.2	4.64	2.19	4.06	1.40
Turn-to-walk	Time	0.16	3.79	7.9	15.0	0.5	2.00	0.47	2.55	0.68
	N steps	0.10	2.18	11.7	4.7	0.1	4.44	1.15	5.33	1.27
	Slope (A-B)	0.04	1.04	10.8	0.5	1.82	2.00	2.07	1.27	1.69
	Slope (B-C)	-0.08	1.18	4.7	4.5	1.7	1.69	1.13	1.17	0.92
Turn-to-sit	RMS	-0.09	1.68	2.5	21.2	1.4	108.02	20.94	98.97	20.19
	Amplitude	-0.13	2.30	1.5	16.3	0.3	183.20	23.33	171.60	26.25
	Slope (D-E)	-0.09	1.34	0.7	20.6	0.07	3.29	1.87	2.80	1.32
	Slope (E-F)	-0.08	1.11	3.74	2.4	0.3	3.15	1.18	2.74	1.32
Stand-to-sit	Time (A-C)	-0.06	1.07	8.4	0.6	0.01	1.38	0.29	1.24	0.35
	Median pitch (A-C)	-0.08	1.42	20.9	13.0	7.0	17.03	8.81	19.39	9.71
	Median pitch (A-B)	-0.07	1.43	18.7	1.3	1.6	12.45	12.06	14.09	9.71
	Max pitch (A-C)	0.13	1.72	8.1	13.4	25.6	87.95	40.36	101.62	35.92
	Mean pitch (A-C)	0.13	2.88	25.1	3.0	1.2	11.61	7.43	18.20	8.80
	Slope pitch (A-B)	0.04	1.64	25.2	0.8	0.2	1.53	0.76	2.09	1.05
	Range pitch (A-B)	0.08	0.86	13.5	32.0	12.1	141.20	34.13	154.88	37.82
	STD pitch (A-C)	0.09	0.96	10.5	18.1	21.9	37.91	11.35	41.72	11.27
	Mean AP (A-C)	-0.07	1.38	0.6	42.9	0.5	0.41	0.14	0.36	0.11
	STD AP (A-C)	0.11	1.75	23.0	27.6	5.4	0.22	0.05	0.25	0.06

### Sit-to-stand phase

For the sit-to-stand phase, 3 of the 23 variables were included. Two of these variables summarize the angular velocity of the movement (pitch signal), in terms of its range and slope. The median of the AP acceleration also was included. Older participants had a larger range, steeper slope and overall a higher acceleration, indicating a movement with a faster change and larger angular movement during standing up and a higher acceleration on average during this period.

### Walking phase

Of the variables related to walking, 6 out of the 14 variables were included in the model: the RMS, gait cycle variability in both the AP and ML directions, the STD step time and the ML acceleration frequency, implying that younger adults had a more variable body sway and more variability between gait cycles and step-times. The variables related the smoothness and regularity of the gait pattern, the mean step time and number of steps, were not included in the model.

### Turn-to-walk phase

For turn-to-walk 4 out of 6 variables were relevant to the PLS model: the slope of the turn phases, the time, and number of steps. During turning while walking, older adults took more time and steps to complete the turn, while the turn of young adults had a steeper slope while turning. A similar number of variables of the turn-to-sit was included, both the slopes and the amplitude and RMS of the angular velocity. During this movement, young adults had more body sway and a larger magnitude of angular velocity. Similarly to the turn-to-walk, young adults had a steeper slope while turning.

### Stand-to-sit phase

Ten out of the total 23 stand-to-sit variables were included in the model. In this phase, in contrast to the other iTUG phases, time was also included. Seven of these 10 variables summarize the angular velocity of the movement (pitch signal). These variables are the mean, median, STD and maximum of the angular velocity during the whole stand-to-sit movement and the median, range and slope of the angular velocity during the first part of the stand-to-sit. The two remaining variables summarize the AP acceleration in terms of the mean and standard deviation of the total stand-to-sit.

Older adults had a faster movement and exhibited on average a higher angular velocity (mean/median) during the stand-to-sit. Their movements also showed a faster change and larger maximum angular velocity and in total a larger range of angular velocity. This was similar to the movement during the sit-to-stand.

During sitting down, young adults had a higher acceleration pattern with a smaller deviation from the mean. With the exception of these results and the higher acceleration of older adults during the sit-to-stand, no variables of the AP acceleration were included in the model of the sit-to-stand and stand-to-sit.

### Classification power to discriminate age groups

**Fig 5:**
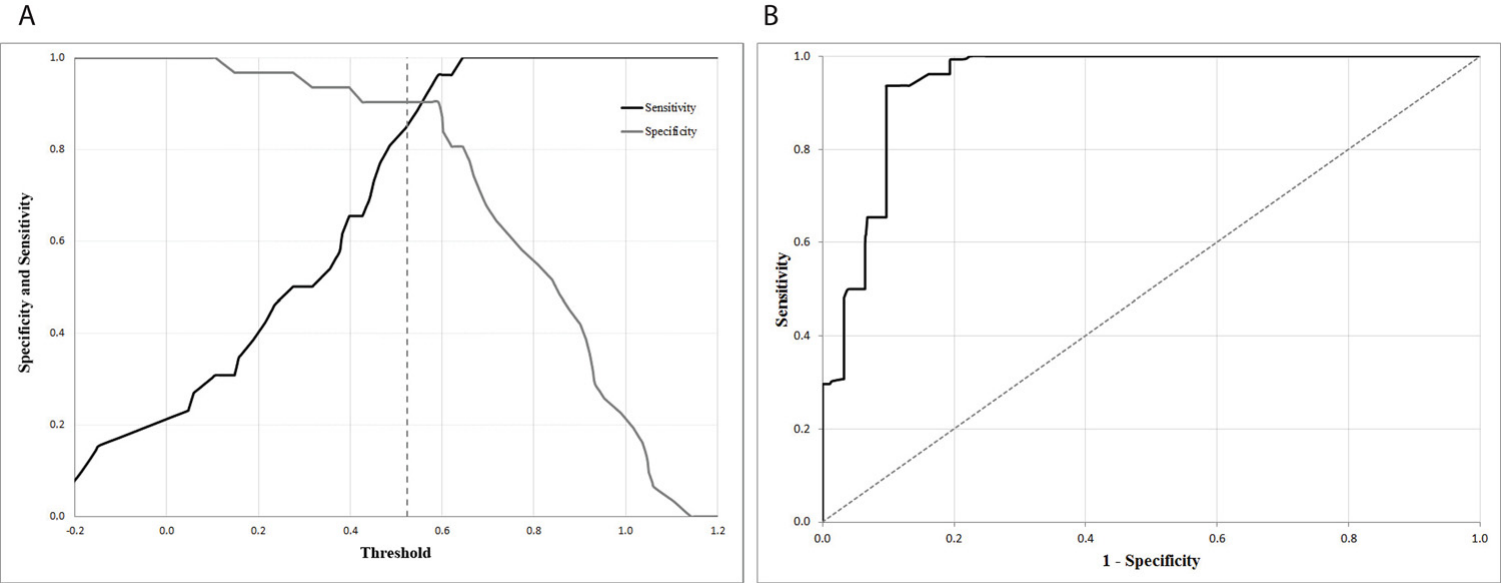
Sensitivity and specificity plots. To determine the optimal cut-off point, sensitivity and specificity are plotted against the threshold (A), the optimal cut-off point is present at 0.52. The sensitivity is plotted against 1—specificity for all cutoff values of the PLS-DA model in the ROC curve (B).

The PLS-DA analysis included the 27 variables identified by the PLS analysis. The model included two latent variables, as for two LVs, the Q2 showed the first peak, before it decreased. 30.6% of the variance in iTUG measures explained 56% of the variance in age groups for these two LVs. The LVs explained respectively 44.1% and 11.5% of the variance in age. The goodness of prediction was 0.38. The analysis had a good accuracy of the classification as indicated by the area under the curve (AUC = 94.7%). Fig 5A shows the ROC curve at the optimal cut-off point, 0.52. The sensitivity and specificity were 90% and 85%, respectively (Fig 5B). These results indicates that 10% or 3 of 26 of the young adults were classified as old and 15% or 5 of 31 of the older adults were classified as young.

## Discussion

The present study addressed two main objectives: 1) which variables of the iTUG are most sensitive to distinguish age effects and 2) what is the classification power of a model based on the variables detected by the first objective. These two objectives were addressed using a multivariate analysis, namely the Partial Least Squares (PLS) analysis. We identified 27 variables of iTUG that predicted age. The subsequent PLS-DA analysis using the 27 identified iTUG variables classified young and old adults with a power of 0.95 and sensitivity and specificity of 90% and 85%. We discuss these results with a perspective on how technology can enrich a widely used clinical test for the purpose of stratifying age groups and patients with high sensitivity and specificity.

### iTUG phase detection

In the present study both an accelerometer and a gyroscope provided data for analyzing the phases of the iTUG in healthy young and older adults. For the phase detection an algorithm that combined a wavelet analysis with a peak detection algorithm was applied, to identify each of the five phases, i.e., sit-to-stand, walk, turn-to-walk, turn-to-sit and stand-to-sit.

Conventionally, TUG performance is scored by a single outcome: total time of execution [8]. In our study, only the time it took to complete the turning phase during the walking period and the duration of the stand-to-sit discriminated older from younger adults. A possible explanation for this result could be that in the current study, we compared healthy participants at different ages ranging from 18 to 75 years of age. Young and older adults completed the iTUG in 14 and 15 s. This could imply that iTUG time has lower sensitivity to differentiate mobility between young and healthy aging old adults. For older adults similar values are reported in other studies (range: 14.3–16.1 s [25]), whereas no reference values for young adults are available.

### Extracted iTUG variables

A combination of the 27 of 72 selected variables consistently identified age-related differences in iTUG performance.

For the sit-to-stand and stand-to-sit phases the variables that revealed differences between young versus old adults were mainly related to the angular velocity (pitch) signal and hardly any differences were detected in the AP accelerations. This is similar to results when comparing healthy older adults to MCI or PD patients [24, 27]. The largest absolute number of variables included in the model, were the 10 variables of the stand-to-sit phase. In contrast, only 3 variables of the sit-to-stand phase were included. Presumably this contrast is related to the lack of reliability of this phase [25]. The data showed that in certain variables there were large differences between the sit-to-stand and stand-to-sit between age groups (Table 2). For example, older adults revealed a larger angular velocity pattern during these two phases and more variability in the angular velocity, an observation perhaps related to the use of greater motor variability during the sit-to-stand to compensate for strength deficits [43]. In another study, older adults have also been shown to be more variable during the sit-to-stand test than young adults [39]. However, this study reported a lower angular velocity during trunk flexion for older adults, while our results show the opposite. A possible explanation for this difference could be that contrary to our study with healthy older adults, the older adults were living in a residential care facility.

Almost all of the selected variables of the turn-to-walk and turn-to-sit were included in the final model. The slope of both turns, indicating fastness and smoothness of turns, was an important discriminating variable and was higher for young adults. For the turn during walking also the larger number of steps during the turn and the longer duration of turning added to the differentiation between the two age groups. For the turn before sitting down, the RMS and amplitude of the yaw signal added to the distinction between the age groups. These outcomes are in line with previous studies that have used the iTUG to distinguish healthy elderly from MCI[24] or PD patients [27], and elderly with an IADL disability [22]. This indicates that the variables during the turning phases of the iTUG not only distinguish age effects, but also pathologies.

The variables related to turns might be even more important in case of pathology considering asymmetric gait in pathologies like stroke, Parkinson’s disease and fallers. Then the direction of the turn will provide additional information and turns should be made in both directions. In our study participants could choose in which direction they made the turn.

Variables of the walking phase in the iTUG that have been reported as being sensitive to discriminate gait of healthy (older) adults from that of patient groups were step regularity, number of steps, duration, IH and Jerk [23, 24, 27]. We found other parameters of walking to be important to the model, namely RMS (ML and AP), gait cycle variability (ML and AP), frequency (ML) and the STD of step time.

The higher gait cycle variability, ML frequency, and STD of step time in young compared to older adults is different from results of previous studies reporting higher step variability in gait of frail elderly and of elderly with fall risk [31, 44, 45]. Our data suggest that adults categorized into a broad age bracket of 18 to 45 years tend to walk with features that resemble a dynamic gait that is somewhat erratic and variable, which is in line with earlier recent findings in this age group. Measures related to smoothness and symmetry of the gait pattern were not included in the model, presumably due to a too low number of steps when walking 7/14 meters. Even when we combined the two walking phases the average number of steps of young and older adults was 17. For smoothness and predictability measures of gait (depending on type of measure), at least 50 steps are required [46].

In summary, the combination of 27 iTUG variables was sensitive to age. In particular variables characterizing gait and the turns were included in the model and these variables were mostly higher in young compared to older adults. In addition, the stand-to-sit phase seemed to differentiate the age groups more accurately than the sit-to-stand. A possible explanation for the larger inclusion of walking-related variables is the fact that gait is a cyclic movement contrary to the discrete transition movement of standing up or sitting down. During walking, older adults may have a more limited set of effective motor solutions compared to young adults, thereby reducing the (goal equivalent) variability [47]. In contrast, during a discrete movement as sitting to standing or vice versa, older adults show more variability [43]. Overall these results underscore the importance of separately assessing the different sub-phases of the iTUG.

### Classification

We deliberately included adults with a wide range of ages to assess changes in iTUG performance over the lifespan of healthy adults. In spite of using non-distinct groups our misclassification rate was only 14%. This result is comparable with the model that was previously developed to distinguish fallers from non-fallers (13%)[25]. Our misclassification rate is lower than a previous model that distinguished two distinct groups, namely healthy older adults from PD patients (22.5%)[23]. This implies that the classification of the current model is similar and possibly better at distinguishing different groups. This could be due to the fact that in the current model 27 variables are included, while the other two models only included three variables. The choice of only a limited number of variables by Palmerini et al.,[25] was based on the statistical model they used, which will lead to an overfitting with a small sample size and large number of parameters. For this reason, we decided to apply a PLS method, because this method is effective in handling relatively small sample sizes with a large number of variables with multi-collinearity [41, 48]. Although the current classification values were good, the model could still be improved. A possible way to improve the classification power (sensitivity/specificity) of the model is to increase the number of age groups with an equal distribution of ages over all groups and/or also increase the number of participants in order to obtain a reference model and/or include more trials in the model to increase the reliability of the individual parameters.

A practical implication of the current model is that the iTUG can be used to successfully distinguish a group of individuals into unique sub-groups (e.g. healthy adults vs. frail adults. A recent trend is to use smart devices, like an iPod or smartphone, as sensors. These devices include embedded accelerometers and gyroscopes. Several studies suggest that these smart devices are reliable to characterize key features of iTUG and gait [40, 49, 50]. This development of the use of smart phones in combination with the development and assessments of models to classify patients, age groups and task effects could have an impact for clinical practice. Smart devices are easy to use, inexpensive, and their use is becoming widespread. Wireless links to an external computer would allow clinicians or researchers to analyze the data without retrieval of the device itself. Also, apps can be programmed for research or clinical practice and the data could then be combined and validated against other data derived from clinical tests [49].

## Conclusion

The current analysis shows that iTUG variables can accurately distinguish healthy young and older adults. A combination of 27 variables, from primarily the turns, walking and stand-to-sit phase was effective to identify iTUG performance in relation to age. The data revealed that young versus older adults executed the TUG with faster and smoother turns and more variable gait cycles and trunk sway during gait. Older adults compared to young adults had a larger angular velocity pattern during the transitions, stand-to-sit and sit-to-stand. Future research should implement the current iTUG analyses for the classification of old adults aging normally and those aging with pathologies. Combined with smart technology, the model could then be used to stratify patients with a high sensitivity and specificity in clinical practice.

## Supporting Information

S1 Dataset Contains all outcome parameters of the data of the first trial from which the first Partial Least Square Model was made. (XLSX)

S2 Dataset File includes the extracted measures of the first PLS model which were used for the Partial Least Square Discriminant Analysis. (XLSX)
